# EPA and DHA engage NRF2 to orchestrate a β-cell resilience circuit suppressing senescence remodeling and sustaining molecular fitness during glucolipotoxic stress

**DOI:** 10.3389/fphys.2026.1948763

**Published:** 2026-10-07

**Authors:** Chaofeng Xing, Fanghong Li, Yan Zou, Cheng Song, Dan Luo, Jian Ruan, Minyi Tang, Qihua Xu, Yunping Mu, Jie Shen, Allan Zijian Zhao

**Affiliations:** 1 The Eighth Affiliated Hospital, Southern Medical University (The First People’s Hospital of Shunde, Foshan), Foshan, Guangdong, China; 2 Laboratory Animal Center, Sun Yat-sen University, Guangzhou, China; 3 GuangDong Engineering Technology Research Center of Metabolic Disorders Interdisciplinary Precision Prevention and Digital Healthcare, The Eighth Affiliated Hospital, Southern Medical University (The First People’s Hospital of Shunde, Foshan), Foshan, Guangdong, China; 4 Center for Reproductive Medicine, the First Affiliated Hospital of Wannan Medical College (Yijishan Hospital of Wannan Medical College), Wuhu, China; 5 Faculty of Health Sciences, University of Macau, Macau, China; 6 The School of Biomedical and Pharmaceutical Sciences, Guangdong University of Technology, Guangzhou, China

**Keywords:** cellular senescence, NRF2, oxidative stress, pancreatic β cell, ω-3 PUFAs

## Abstract

**Introduction:**

Preservation of pancreatic β cells under sustained glucolipotoxicity requires more than limiting cell death, because surviving cells retain stress-associated molecular abnormalities. We tested whether EPA and DHA act through an NRF2-dependent protective program in β-cell models.

**Methods:**

MIN6 cells and primary mouse islets were exposed to palmitate plus high glucose with or without EPA, DHA, or EPA+DHA. NRF2 nuclear localization, glutathione homeostasis, oxidative DNA damage, apoptosis, senescence markers, and β-cell maturity proteins were assessed, and NRF2 knockdown was used to test NRF2 dependency. Male C57BL/6J mice with established HFD/STZ diabetes received EPA, DHA, or EPA+DHA; glycemic indices and pancreatic β-cell markers were evaluated.

**Results:**

EPA and DHA increased nuclear NRF2, restored glutathione homeostasis, and reduced oxidative DNA damage, apoptotic markers, and senescence markers in both MIN6 cells and primary islets. NRF2 knockdown partially attenuated these responses, indicating that NRF2 contributes to but is not the sole mediator of the *in vitro* protection. In HFD/STZ mice, EPA/DHA improved glycemic indices, reduced oxidative-stress and apoptotic markers, and preserved insulin-positive β-cell area.

**Discussion:**

These findings identify an NRF2-sensitive program through which EPA and DHA preserve antioxidant capacity, survival, and maturity features of β cells under glucolipotoxic stress. Partial attenuation by NRF2 knockdown and the systemic nature of the HFD/STZ model indicate that additional NRF2-independent mechanisms contribute, and that β-cell specific NRF2 deletion will be needed to establish β-cell autonomous causality *in vivo*.

## 1 Introduction

Type 2 diabetes mellitus (T2DM) develops when pancreatic β cells can no longer sustain insulin demand imposed by systemic insulin resistance. This failure is often quantified as a loss of β-cell mass, yet cell survival alone does not define successful adaptation. β cells that escape death may remain insulin positive while entering dysfunctional states characterized by persistent damage signaling, senescence-associated remodeling, metabolic immaturity, or erosion of the transcriptional network that supports mature β-cell function (Weir and Bonner-Weir, 2004; Guo et al., 2013; Lytrivi et al., 2020). The biological outcome of metabolic stress is therefore not binary. Glucolipotoxic β cells may die, retain adaptive competence, or survive in a state that contributes little to effective insulin secretion and may adversely influence neighboring islet cells. Preserving β-cell mass is consequently meaningful only when survival is the molecular integrity of the surviving cell population.

Oxidative stress is positioned at the center of this divergence in cell fate. Chronic exposure to high glucose and saturated fatty acids increases mitochondrial substrate pressure, reactive oxygen species (ROS) production, lipid peroxidation, and oxidative DNA damage (Lenzen, 2008; Poitout and Robertson, 2008). Because β cells combine high metabolic and secretory activity with relatively limited antioxidant capacity, these disturbances can rapidly exceed their redox reserve (Lenzen, 2008). Severe or unresolved injury activates mitochondrial apoptosis, whereas sublethal damage can sustain p53-p21 and p16-RB signaling, senescence-associated β-galactosidase activity, inflammatory cytokine release, and disruption of mature β-cell gene expression (Aguayo-Mazzucato et al., 2019; Gorgoulis et al., 2019). These outcomes are related but not interchangeable. Apoptosis removes β cells, stress-associated and identity-disruptive responses may leave surviving cells with impaired islet function. This distinction creates a central therapeutic challenge: a protective intervention must prevent cell loss without merely converting lethal injury into dysfunctional survival.

Eicosapentaenoic acid (EPA) and docosahexaenoic acid (DHA) are plausible regulators of this balance because their biological actions extend beyond passive incorporation into cellular lipids. These ω-3 polyunsaturated fatty acids (ω-3 PUFAs) influence membrane organization, lipid-derived signaling, inflammatory tone, and cellular redox responses (Calder, 2010). Experimental studies have shown that EPA and DHA can reduce oxidative injury and apoptosis in insulin-secreting cells and pancreatic islets (Wei et al., 2010; Lucena et al., 2015; Graciano et al., 2016). However, evidence from human randomized trials has not consistently demonstrated improved diabetes risk or glycemic outcomes with long-chain ω-3 PUFAs supplementation (Brown et al., 2019). Those observations support biological activity in experimental systems but do not establish clinical efficacy or indicate that EPA and DHA are functionally interchangeable. Whether ω-3 PUFAs influence the molecular state of surviving β cells, in addition to limiting injury, remains to be clarified.

Nuclear factor erythroid 2-related factor 2 (NRF2) provides a mechanistic basis for resolving this problem. Upon release from Kelch-like ECH-associated protein 1 (KEAP1)-dependent repression, NRF2 accumulates in the nucleus and activates antioxidant response element-dependent genes, including Hmox1, NQO1, SOD1, GPX4, and Slc7a11 (Yamamoto et al., 2018; Cuadrado et al., 2019). Together, these effectors support glutathione availability, ROS detoxification, and resistance to lipid peroxidation. Importantly, NRF2 is already recognized as a broad regulator of functional β-cell mass, with reported roles in redox homeostasis, survival, proliferation, and insulin-secretory function under metabolic stress (Haidery et al., 2025). Genetic studies in β cells further show that NRF2 activity influences their capacity to withstand metabolic and oxidative injury (Fu et al., 2015; Baumel-Alterzon et al., 2021). Accordingly, the present study does not propose a previously unrecognized role for NRF2 in β-cell preservation. Rather, it examines whether an EPA/DHA-associated NRF2 response is accompanied by coordinated changes in oxidative injury, apoptotic signaling, senescence-associated stress markers, and β-cell identity-associated proteins under glucolipotoxic conditions. Here, we examined whether EPA and DHA engage NRF2 to coordinate these distinct stress outcomes under glucolipotoxic conditions. Using MIN6 cells and primary mouse islets, we examined NRF2 activation alongside redox injury, mitochondrial apoptosis, senescence-associated stress signaling and inflammatory output, and proteins linked to mature β-cell identity. *NRF2* loss-of-function analysis was used to determine whether NRF2 contributes to selected EPA/DHA-associated molecular responses rather than merely associated with it. We then examined whether ω-3 PUFAs supplementation reproduced this protective pattern in male HFD/STZ mice exposed to combined insulin resistance and direct β-cell injury. Because the HFD/STZ model incorporates systemic metabolic changes as well as pancreatic injury, the *in vivo* experiments were not designed to establish β-cell-autonomous NRF2 causality. Our findings therefore assess an NRF2-sensitive component of the cellular response to EPA and DHA, rather than defining NRF2 as the sole mechanistic link between ω-3 PUFAs and β-cell protection.

## 2 Materials and methods

### 2.1 Reagents

EPA (D8768, Sigma-Aldrich, CA, USA) and DHA (E8768, Sigma-Aldrich) were dissolved in DMSO to prepare a working stock solution of approximately 100 mM. Palmitic acid (PA, P5585, Sigma-Aldrich) was dissolved in 0.1 M NaOH at 70 °C to generate a 100 mM stock solution. A 5% (w/v) fatty-acid-free BSA (FFA-BSA, ST2254, Beyotime, shanghai, China) solution was prepared in 150 mM NaCl with warming to 50 °C. The PA-BSA conjugate was formed by dropwise addition of the PA stock into the pre-warmed BSA solution under vortexing to achieve a 5:1 molar ratio (PA: BSA), followed by incubation at 37 °C for 1 hour with gentle agitation. A control BSA solution was prepared identically using vehicle (0.1 M NaOH) without PA.

### 2.2 Animals and models

Animal model and experimental design. A murine model of T2DM was established in male C57BL/6J mice (7 weeks old, GemPharmatech Co., Ltd.) through combined dietary and pharmacological interventions. To induce insulin resistance, mice were fed a high-fat diet (HFD, 60% kcal from fat, D12492, Research Diets) for 4 weeks, while control animals received a standard chow diet. Subsequently, HFD-fed mice received a single intraperitoneal injection of streptozotocin (STZ, 100 mg/kg in ice-cold citrate buffer, S0130, Sigma) to impair β cell function. The diabetic phenotype was confirmed by measuring non-fasting blood glucose levels on two consecutive weeks, and only mice with sustained hyperglycemia (>16.7 mmol/L) were included in the study (Sim et al., 2023). Following successful model induction, animals were systematically allocated into the following groups (*n* = 6/group): a normal control (NC) group receiving saline; an HFD/STZ model group receiving the corn oil vehicle (H+S), and HFD/STZ groups treated with docosahexaenoic acid (DHA, 90%, OMEGOR), eicosapentaenoic acid (EPA, 97%, OMEGATREASURE), or their combination (E+D, EPA: DHA = 11:8, 95%, OMEGATREASURE). All compounds were administered via oral gavage at a dose of 3 g/kg/day. All experimental procedures were approved by the Laboratory Animal Ethics Committee of The Eighth Affiliated Hospital, Southern Medical University (Approval No. SDYY-XZF-21-2408-010) and conducted in accordance with institutional guidelines.

### 2.3 Cells culture and treatment

MIN6 cells (CC-Y2103, Shanghai Enzyme Research Biotechnology) were cultured in high-glucose Dulbecco’s Modified Eagle Medium (DMEM, 12800017, Gibco), supplemented with 10% FBS, and 50 μM β-mercaptoethanol (PB180633, procell, Wuhan, China). Cells were maintained in a humidified incubator at 37 °C with 5% CO_2_. MIN6 cells were then exposed to media containing normal glucose (NC, 5.5 mM) as a control or high glucose (HG, 25 mM) + palmitic acid (PA, 0.5 mM) for 24 hours. In experiments to assess the protective effects of ω-3 PUFAs, MIN6 cells were pretreated for 24 hours with 50 μM EPA, DHA, or EPA/DHA (25 μM each), followed by co-treatment with 25 mM glucose and 0.5 mM PA (Zhang et al., 2021) for an additional 24 hours. Cells in the normal control (NC) group were cultured in 5.5 mM glucose throughout.

### 2.4 Cell counting kit-8 assay

MIN6 cells were pretreated with EPA, DHA, or EPA+DHA (25 μM each for the combination) at 10, 25, 50, 75, or 100 μM for 24 h, followed by co-treatment with 25 mM glucose and 0.5 mM PA for an additional 24 h in the continued presence of the corresponding fatty acid. The PA concentration was held constant across all groups. Cell viability was assessed using the CCK-8 assay (E606335, Sangon Biotech, Shanghai, China). Relative to the PA/HG group, EPA, DHA, and EPA+DHA each improved viability across the tested range, with the greatest mean protective response at 50 μM, higher concentrations (75-100 μM) did not provide additional benefit. Therefore, 50 μM EPA or DHA​ was selected as the working concentration for subsequent experiments. For the EPA+DHA condition, the 50 μM total concentration comprised 25 μM EPA and 25 μM DHA.

### 2.5 Isolation of mouse pancreatic islets and treatment

Pancreatic islets were isolated from mice following institutional guidelines. Mice were anesthetized with Avertin (tribromoethanol, T161626, Aladin), the common bile duct was cannulated and perfused with cold collagenase P (1mg/mL in HBSS, 11249002001, sigma) to inflate the pancreas, which was then excised. Digestion proceeded at 37 °C for 10 minutes with agitation and was terminated with cold HBSS include 10% FBS. The crude digest was washed, Islets were purified using a discontinuous density gradient centrifugation. The gradient was formed with Histopaque-1119 (11191, Sigma) and Histopaque-1077 (10771, Sigma). After centrifugation, the islets were collected from the interface and hand-picked under a stereomicroscope (Xing et al., 2023). Following collection from the interfaces, islets were hand-picked for purity and cultured overnight in RPMI-1640 medium (31800022, Gibco, Grand Island, NY, USA) supplemented with 10% fetal bovine serum (FBS, 164210-50, Procell), and 50 μM β-mercaptoethanol. Isolated mouse pancreatic islets were pretreated for 24 h with EPA (50 μM), DHA (50 μM), or their combination (E+D; 25 μM EPA plus 25 μM DHA), followed by a further 24 h of co-treatment with palmitic acid (PA, 0.5 mM) and high glucose (25 mM) in the continued presence of the corresponding fatty acid treatment (Zhang et al., 2021). Cells in the normal control (NC) group were cultured in 11.11 mM glucose throughout.

### 2.6 Knockdown of NRF2 in MIN6 with shRNA

To establish a stable NRF2-knockdown MIN6 cell line, a short hairpin RNA (shRNA) targeting Nrf2 was cloned into a pSLenti-U6 vector. The recombinant plasmid was then packaged into lentiviruses, which were used to transduce MIN6 cells. After 48 hours of culture in complete DMEM, transduced cells were selected with puromycin (1 µg/mL) for 48 hours to generate a stable pool. The knockdown efficiency was validated by Western blotting. As shown in **Figures 1A, B**, shNRF2 transduction resulted in a robust knockdown of NRF2 protein expression compared to the control, and the most effective construct was selected for subsequent experiments.

**Figure 1:**
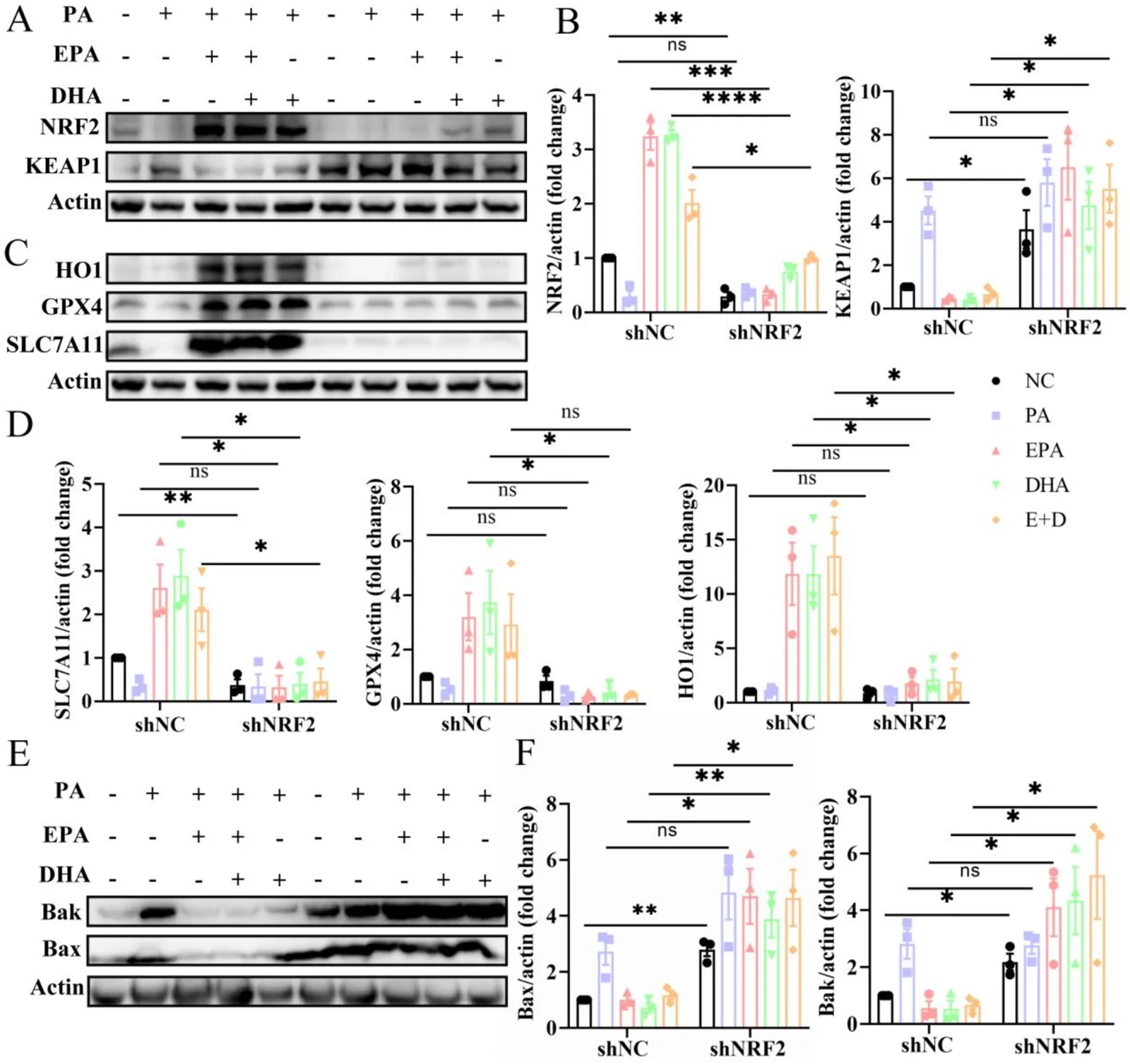
NRF2 knockdown attenuates the effects of ω-3 PUFAs on selected antioxidant and apoptotic markers in MIN6 cells. MIN6 cells were transfected with shNRF2 or control shRNA, followed by treatment with ω-3 PUFAs and palmitic acid (PA, 50 μM). **(A, B)** Western blot analysis and quantification of NRF2 and its endogenous inhibitor KEAP1 (*n* = 3). **(C, D)** Expression levels of key NRF2 downstream antioxidant proteins (HO-1, SLC7A11, GPX4) (*n* = 3). **(E, F)** Analysis of pro-apoptotic proteins Bak and Bax, demonstrating the dependence of ω-3 PUFAs anti-apoptotic effect on NRF2 (*n* = 3). Data are expressed as the mean ± SEM from three independent biological replicates. Two-way ANOVA with shRNA status and treatment as factors, followed by Sidak’s multiple-comparisons test. *P < 0.05, **P < 0.01, ***P < 0.001, ****P < 0.0001, *ns* indicates no significant difference.

### 2.7 Senescence associated β-galactosidase activity assay

Senescence-associated β-galactosidase (SA-β-gal) activity was assessed using a commercial staining kit (Servicebio, Wuhan, China) following the manufacturer’s instructions. Briefly, Frozen section cultured in a 6-well plate were fixed with fixative solution for 15 minutes at room temperature and washed three times with phosphate-buffered saline (PBS). Subsequently, a staining solution composed of 94% staining solution A, 1% staining solution B (both provided in the kit), and 5% X-gal was added to each well. The cells were incubated with the staining solution in the dark at 37 °C overnight (approximately 12 hours). Images were acquired using a Leica microscope.

### 2.8 Immunofluorescence staining

Pancreatic tissue samples of mice were fixed, paraffin-embedded, and sectioned. After deparaffinization and antigen retrieval in citrate buffer, sections were permeabilized with 0.1% Triton X-100 and blocked with 5% BSA. Subsequently, sections were incubated with a primary antibody overnight at 4 °C, followed by a fluorophore-conjugated secondary antibody for 1 hour at room temperature. Nuclei were counterstained with DAPI, and slides were mounted for imaging under a fluorescence/confocal microscope. The antibodies used were as follows: anti-Insulin (4590, Cell Signaling Technology), anti-glucagon (G2654, sigma), anti-P16 (ab189034, Abcam), anti-NRF2 (sc-365949, Santa Cruz Biotechnology), secondary antibody conjugated to Alexa Fluor^®^ 555, anti-rabbit secondary antibody conjugated to Alexa Fluor^®^ 488, anti-mouse secondary antibody conjugated to Alexa Fluor^®^ 488 or anti-rabbit secondary antibody conjugated to Alexa Fluor^®^ 647 (Cell Signaling Technology).

### 2.9 Measurement of reactive oxygen species, glutathione and 8-hydroxy-2’-deoxyguanosine

Intracellular reactive oxygen species (ROS) levels were quantified using the fluorescent probe 2’,7’-dichlorodihydrofluorescein diacetate (DCFH-DA; S0033, Beyotime). In brief, MIN6 cells were loaded with 10 μM DCFH-DA and incubated at 37 °C for 20 minutes, followed by three washes with phosphate-buffered saline (PBS). The fluorescence intensity, indicative of intracellular ROS, was immediately measured using fluorescence microscopy and normalized to that of the control group. The cellular glutathione (GSH) content was determined with a commercial assay kit (S0052, Beyotime) and expressed as nanomoles per milligram of protein (nmol/mg protein). The extent of oxidative DNA damage was assessed by measuring the level of 8-hydroxy-2’-deoxyguanosine (8-OHdG) using a specific ELISA kit (D751009, Sangon Biotech), with concentrations reported as ng/mL in serum and ng/mg protein in cellular lysates, respectively.

### 2.10 Measurement of total antioxidant capacity, superoxide dismutase activity, catalase activity, and malondialdehyde

T-AOC, SOD activity, CAT activity, and MDA content were measured in mouse serum using commercial colorimetric assay kits according to the manufacturers’ instructions. T-AOC was determined by the FRAP method (S0116, Beyotime) and expressed as mmol FeSO_4_ equivalent/mg protein. SOD activity was measured by the WST-8 method (S0101, Beyotime) and expressed as U/mg protein. CAT activity was assessed by the ammonium molybdate method (S0058, Beyotime) and expressed as U/mg protein. MDA content was determined by the TBARS method (D751009, Sangon Biotech) and expressed as nmol/mg protein for cell lysates or nmol/mL for serum.

### 2.11 Insulin tolerance test

Insulin tolerance was assessed in mice fasted for 6 h. The animals were injected intraperitoneally with human recombinant insulin. To account for varying insulin sensitivity, recombinant insulin was administered intraperitoneally at 0.75 U kg⁻¹ body weight to all groups. Blood glucose levels were determined from tail vein blood samples at 0 (pre-injection), 15, 30, 60, 90, and 120 minutes after injection using a glucometer.

### 2.12 Intraperitoneal glucose tolerance test

An intraperitoneal glucose tolerance test was performed on mice fasted for 6 hours. Baseline glycemia (time 0) was measured prior to the administration of a glucose load (2 g/kg, i.p.). Subsequent blood glucose levels were monitored at 15, 30, 60, 90, and 120min post-injection. The results are plotted as a glucose curve, and the area under the curve (AUC) is calculated to provide a quantitative measure of glucose tolerance.

### 2.13 Western blot

MIN6 cells were lysed with Radio-Immunoprecipitation Assay (RIPA) buffer (P001313, Beyotime) to obtain total protein extracts. Prior to immunoblotting, the protein concentration of each sample was determined in duplicate using a bicinchoninic acid (BCA) assay kit (23225, Thermo Fisher Scientific) to ensure equal loading. Western blot analysis was then conducted following a standard protocol (Xing et al., 2023). The antibodies used were as follows: anti-GAPDH (5174, Cell Signaling Technology), anti-p21 (2947, Cell Signaling Technology), anti-cleaved-PARP (5625, Cell Signaling Technology), anti-p-Rb (8180, Cell Signaling Technology), anti-Rb (9309, Cell Signaling Technology), anti-Bax (2772, Cell Signaling Technology), anti-Bak (6947, Cell Signaling Technology), anti-p53 (2524, Cell Signaling Technology), anti-HO-1 (43966, Cell Signaling Technology), anti-NQO1 (62262, Cell Signaling Technology), anti-SLC7A11 (12691, Cell Signaling Technology), anti-P16 (80772, Cell Signaling Technology), and anti-KEAP1 (8047, Cell Signaling Technology), anti-β-Actin (A2228, sigma), anti-NRF2 (sc-365949, Santa Cruz Biotechnology), anti-β-Tubulin (GB15140, Servicebio), anti-Bcl-2 (ab182858, abcam), anti-GPX4 (ab125066, abcam), anti-Pax4 (DF2605, Affinity Biosciences), anti-Pdx1 (5679, Cell Signaling Technology), anti-mafa (NB400-137, Novus Biologicals), NeuroD1 (7019, Cell Signaling Technology), goat anti-mouse-HRP (115-035-003, Jackson Immuno-Research) and goat anti-rabbit HRP (111-035-003, Jackson Immuno-Research).

### 2.14 Measurement of interleukin-6, tumor necrosis factor-α and C-X-C motif chemokine ligand 1

Mouse serum was collected for cytokine measurement. The concentrations of interleukin-6 (IL-6; MM-0163M1, Enzyme-linked Biotechnology), tumor necrosis factor-α (TNF-α; MM-0132M1, Enzyme-linked Biotechnology), and C-X-C motif chemokine ligand 1 (CXCL1; MM-43835M1, Enzyme-linked Biotechnology) were determined using commercial enzyme-linked immunosorbent assay kits according to the manufacturer’s instructions. Briefly, standards and samples were added to pre-coated plates and incubated sequentially with the corresponding detection reagents and substrate solution. Absorbance was measured using a microplate reader, and cytokine concentrations were calculated from standard curves.

### 2.15 Statistical analysis

Data were presented as the mean ± SEM from three independent biological replicates. For cell-based assays, each biological replicate represents an independently performed experiment, with technical replicate wells measured within each experiment. For animal-derived samples, biological replicates represent individual mice or independently isolated islet preparations, as indicated. The statistical significance was assessed by Student’s *t*-test or by analysis by one-way variance (ANOVA) with Sidak’s multiple comparisons test in Prism software (GraphPad Prism 8.0). Nrf2 knockdown experiments were analyzed by two-way ANOVA with shRNA status and treatment as factors, followed by Sidak’s multiple-comparisons test. A value of *P* < 0.05 was considered statistically significant.

## 3 Results

### 3.1 ω-3 PUFAs protect pancreatic β cell from glucolipotoxicity by activating the NRF2-mediated antioxidant pathway

To investigate the protective effects of ω-3 PUFAs against glucolipotoxicity, MIN6 cells and isolated mouse pancreatic islets were exposed to palmitic acid (PA) and high glucose (HG). Based on CCK-8 concentration screening (**Supplementary Figure S1**), EPA and DHA were used at 50 μM, the combined-treatment group received 25 μM EPA plus 25 μM DHA (E+D). MIN6 cells and islets were pretreated with the indicated ω-3 PUFAs for 24 h, followed by a further 24 h of PA/HG exposure in the continued presence of the corresponding treatment. Relative to the PA/HG model group, EPA, DHA, and E+D reduced intracellular ROS and 8-hydroxy-2′-deoxyguanosine (8-OHdG) levels and restored glutathione (GSH) content in MIN6 cells, indicating attenuation of glucolipotoxic oxidative injury (Li et al., 2025). (**Figures 2A–E**), indicating a strong protective effect from PA-induced oxidative stress. To elucidate the molecular mechanism underlying this antioxidant effect, we examined the NRF2/HO1 pathway, a central regulator of the cellular antioxidant response (Carlson et al., 2024). Western blotting of whole-cell lysates showed an increased total abundance NRF2 and elevated levels of its downstream targets, including heme oxygenase-1 (HO-1), NAD(P)H quinone dehydrogenase 1 (NQO1), superoxide dismutase 1 (SOD1) (Li et al., 2024), and the cystine/glutamate antiporter subunit SLC7A11 (a critical subunit for GSH synthesis) (Wu et al., 2025) in MIN6 cells. Concomitantly, ω-3 PUFAs treatment reduced the expression of KEAP1, an endogenous negative regulator of NRF2 in PA-treated MIN6 cells (**Figures 2F, G**). Immunofluorescence was used separately to assess NRF2 subcellular localization. EPA, DHA, and their combination (E+D), increased the proportion of insulin-positive MIN6 cells and islet cells displaying nuclear NRF2 staining (**Figures 2H, J**), as well as isolated mouse pancreatic islets (**Figures 2I, K**). Thus, Western blotting and immunofluorescence assays respectively report total cellular NRF2 abundance and nuclear NRF2 localization. Together, these findings show that EPA and DHA are associated with reduced glucolipotoxic oxidative injury and an NRF2-associated antioxidant response in MIN6 cells and primary mouse islets.

**Figure 2:**
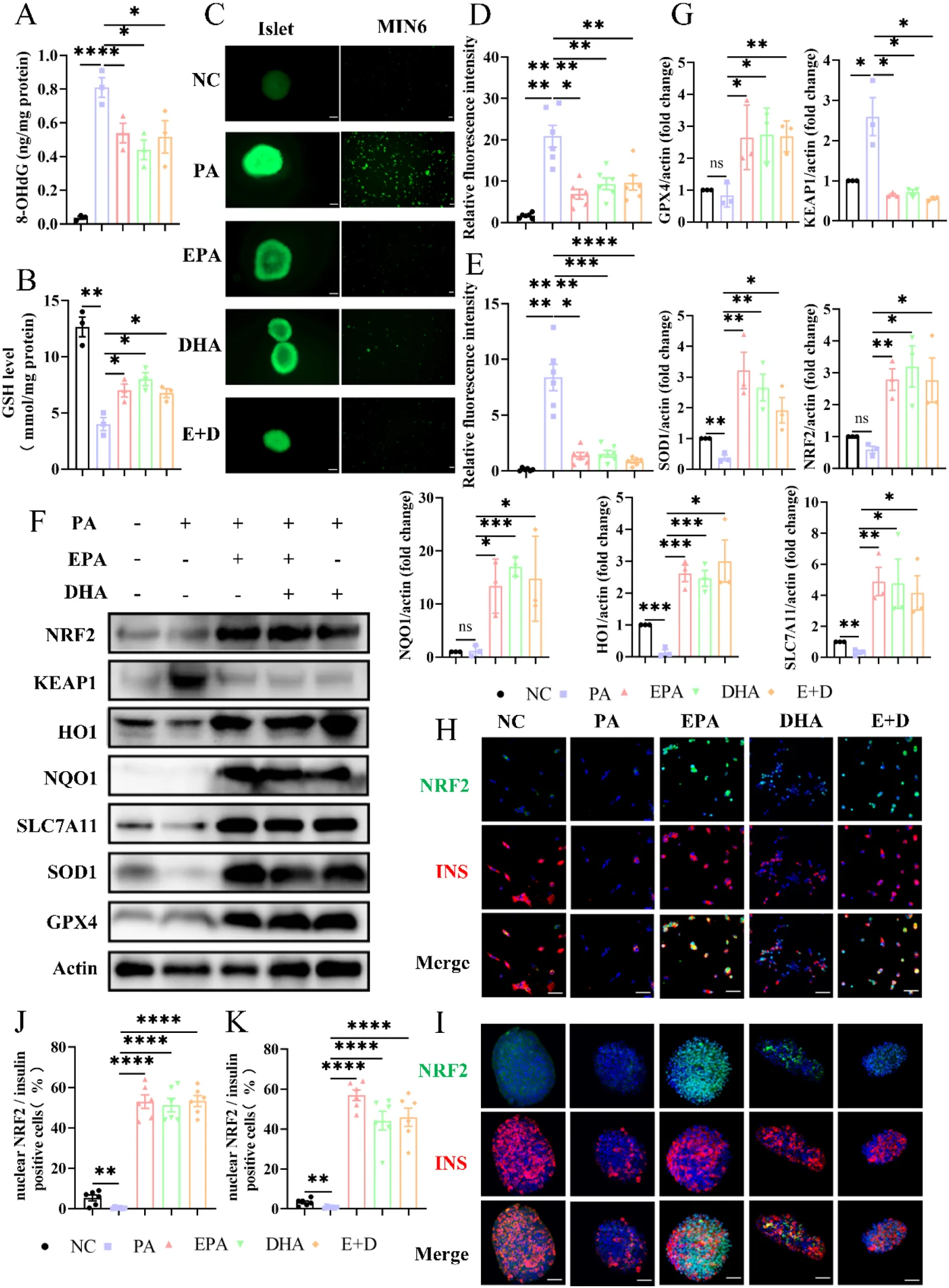
ω-3 PUFAs mitigate high glucose and palmitic acid (PA)-induced oxidative stress in β cell *in vitro*. MIN6 cells and isolated mouse pancreatic islets were pretreated with EPA, DHA, or EPA+DHA (1:1) for 24 h, followed by a 24h PA (0.5 mM)/HG (25 mM) challenge in the continued presence of the corresponding treatment. **(A–C)** Levels of oxidative stress markers: 8-OHdG, glutathione (GSH), and intracellular reactive oxygen species (ROS, green). **(D)** Statistical analysis of ROS levels in pancreatic islets (DCFH-DA staining) from **(C)**. **(E)** Statistical analysis of ROS levels in MIN6 cells (DCFH-DA staining) from **(C)**. **(F, G)** Western blot analysis of key proteins in the antioxidant defense pathway, including NRF2, KEAP1, HO-1, NQO1, SOD1, GPX4, and SLC7A11 (*n* = 3). **(H, I)** Immunofluorescence staining showing the localization of DAPI (blue), NRF2 (green) and insulin (red) in **(H)** MIN6 cells and **(I)** pancreatic islets. **(J)** Statistical analysis of NRF2 and insulin co-staining intensity in MIN6 cells from **(H)**. **(K)** Statistical analysis of NRF2 and insulin co-staining intensity in pancreatic islets from **(I)**. Data are expressed as the mean ± SEM from three independent biological replicates. For cell-based assays, each biological replicate represents an independently performed experiment using isolated islet preparations, as indicated. Statistical analysis was performed using one-way ANOVA followed by Sidak’s multiple-comparisons test. *P < 0.05, **P < 0.01, ***P < 0.001, ****P < 0.0001. Scale bar: 50 μm.

### 3.2 ω-3 PUFAs attenuate glucolipotoxicity-associated apoptotic signaling

Given the association between glucolipotoxic oxidative injury and β-cell apoptosis, we next examined whether the antioxidative action of EPA or DHA translates into protection against β cell apoptosis. To this end, MIN6 cells or mouse pancreatic islets were pretreated with EPA, DHA, or their combination (E+D) for 24h, followed by exposure to PA (0.5 mM) and HG (25 mM) for an additional 24 h in the continued presence of the corresponding treatment and co-treated with PA (0.5 mM) and HG (25 mM) for 24h. TUNEL/insulin co-staining showed that PA/HG increased the proportion of TUNEL-positive insulin-positive cells in MIN6 cultures and mouse islets. EPA, DHA, and E+D reduced this proportion relative to the PA/HG group (**Figures 3C–F**). (**Figures 3C–F**). Western blot analysis of apoptosis-related proteins in MIN6 cells revealed that ω-3 PUFAs attenuated the PA/HG-associated changes in apoptosis-related proteins. Accordingly, ω-3 PUFAs significantly decreased the expression of the pro-apoptotic proteins Bax and Bak, while enhancing the levels of the anti-apoptotic proteins Bcl-2 and Bcl-XL (Sitthisuk et al., 2024). This change in Bcl-2 family proteins was accompanied by reduced cleaved PARP abundance (**Figures 3A, B**), indicating suppression of the mitochondrial apoptotic pathway. Consistent with attenuation of apoptosis-associated signaling under glucolipotoxic conditions.

**Figure 3:**
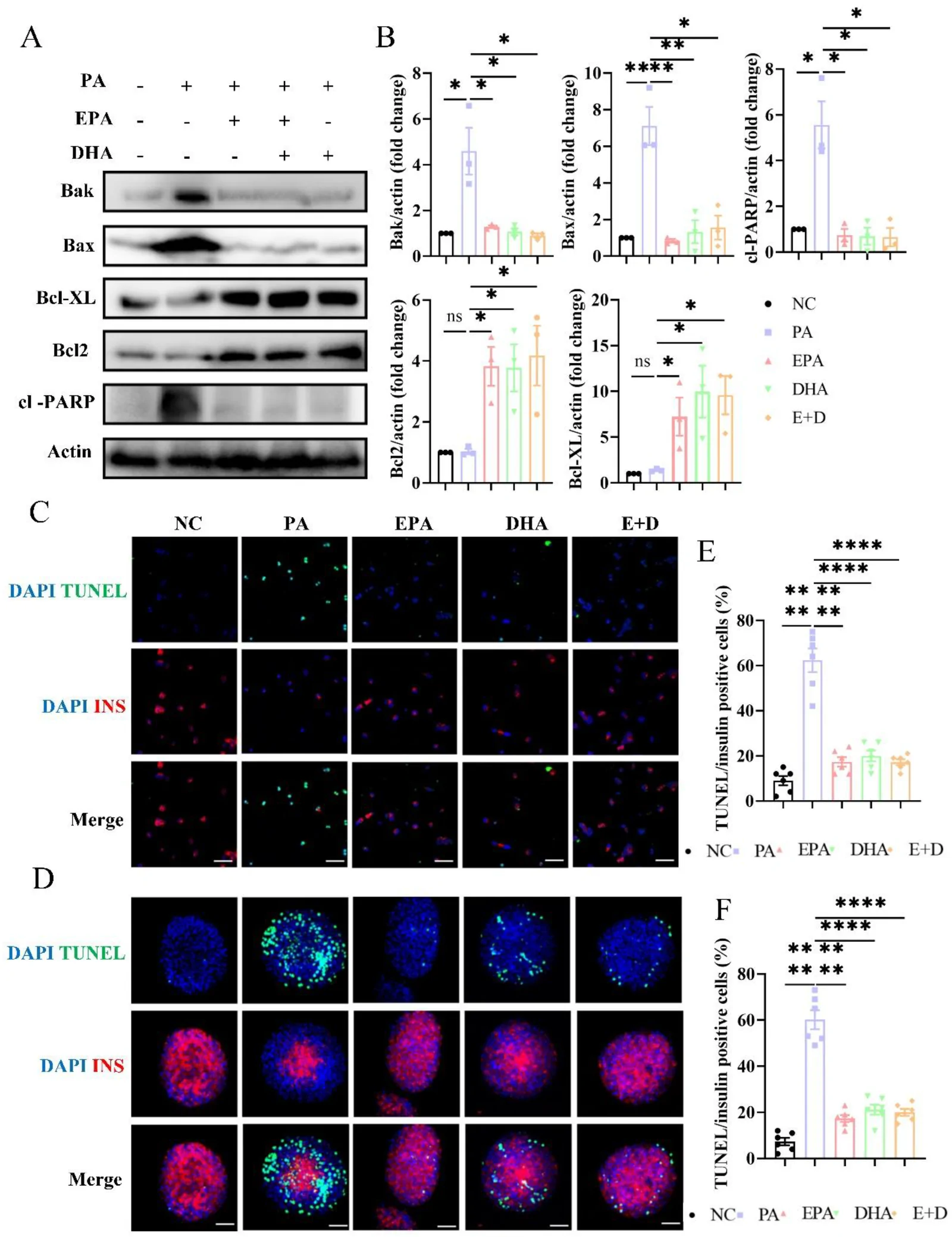
ω-3 PUFAs suppress glucolipotoxicity-induced mitochondrial apoptosis *in vitro*. MIN6 cells and isolated mouse pancreatic islets were pretreated with EPA, DHA, or EPA+DHA (1:1) for 24 h, followed by a 24h PA (0.5 mM)/HG (25 mM) challenge in the continued presence of the corresponding treatment. **(A, B)** Western blot analysis of key apoptosis-related proteins, including Bcl-2, Bcl-XL, Bax, Bak, and cleaved PARP (cl-PARP) in MIN6 cells (*n* = 3). **(C, D)** Assessment of apoptosis by DAPI (blue), TUNEL (green) and insulin (red) co-staining in **(C)** MIN6 cells and **(D)** pancreatic islets. **(E)** Statistical analysis of TUNEL and insulin co-staining intensity in MIN6 cells from **(C)**. **(F)** Statistical analysis of TUNEL and insulin co-staining intensity in pancreatic islets from **(D)**. Data are expressed as the mean ± SEM from three independent biological replicates. For cell-based assays, each biological replicate represents an independently performed experiment using isolated islet preparations, as indicated. Statistical analysis was performed using one-way ANOVA followed by Sidak’s multiple-comparisons test. *P < 0.05, **P < 0.01, ****P < 0.0001. Scale bar: 50 μm.

### 3.3 ω-3 PUFAs attenuate senescence-associated stress markers and preserve a maturity-associated protein profile under glucolipotoxic stress

We next assessed senescence-associated stress markers in glucolipotoxic MIN6 cells. Western blot analysis of MIN6 cells under glucolipotoxic (PA + HG) conditions revealed that ω-3 PUFAs treatment significantly downregulated the expression of p16, p21, and p53. Concomitantly, we observed a marked increase in the phosphorylation level of the retinoblastoma (RB) protein (**Figures 4A, B**). These results indicate attenuation of p53/p21/p16-associated stress signaling under the acute glucolipotoxic conditions examined. Immunofluorescence further confirmed a pronounced reduction of P16 in MIN6 cells (**Figures 4C**; **Supplementary Figure S2A**) and in mouse pancreatic islets (**Figures 4D**; **Supplementary Figure S2B**). Senescence-associated β-galactosidase (Potdar et al., 2025) activity was also significantly lowered by ω-3 PUFAs treatment (**Figures 4E**; **Supplementary Figures S2C**, **S1D**). Although EPA, DHA, and E+D reduced SA-β-gal-positive area relative to the PA/HG group, staining was not fully restored to the normal-control level. Together, these data show that ω-3 PUFAs attenuated apoptosis-associated and senescence-associated stress markers; they do not establish reversal of an irreversibly established senescent state or preservation of β-cell function. Finally, we examined whether this stress protection was accompanied by preservation of β-cell identity-associated proteins. PA induced LDHA, a protein associated with loss of special β-cell metabolic features, while reducing Mafa, NeuroD1, Pax4, and Pdx1 (Lin et al., 2026) (**Figures 4G, H**). EPA, DHA, and E+D suppressed LDHA induction and partially restored these β-cell identity-associated transcription factors. Together with the oxidative-stress and apoptosis-associated findings, these data indicate that ω-3 PUFAs were associated with coordinated changes in redox balance, apoptotic processes, senescence-associated stress, and maturity-associated protein endpoints.

**Figure 4:**
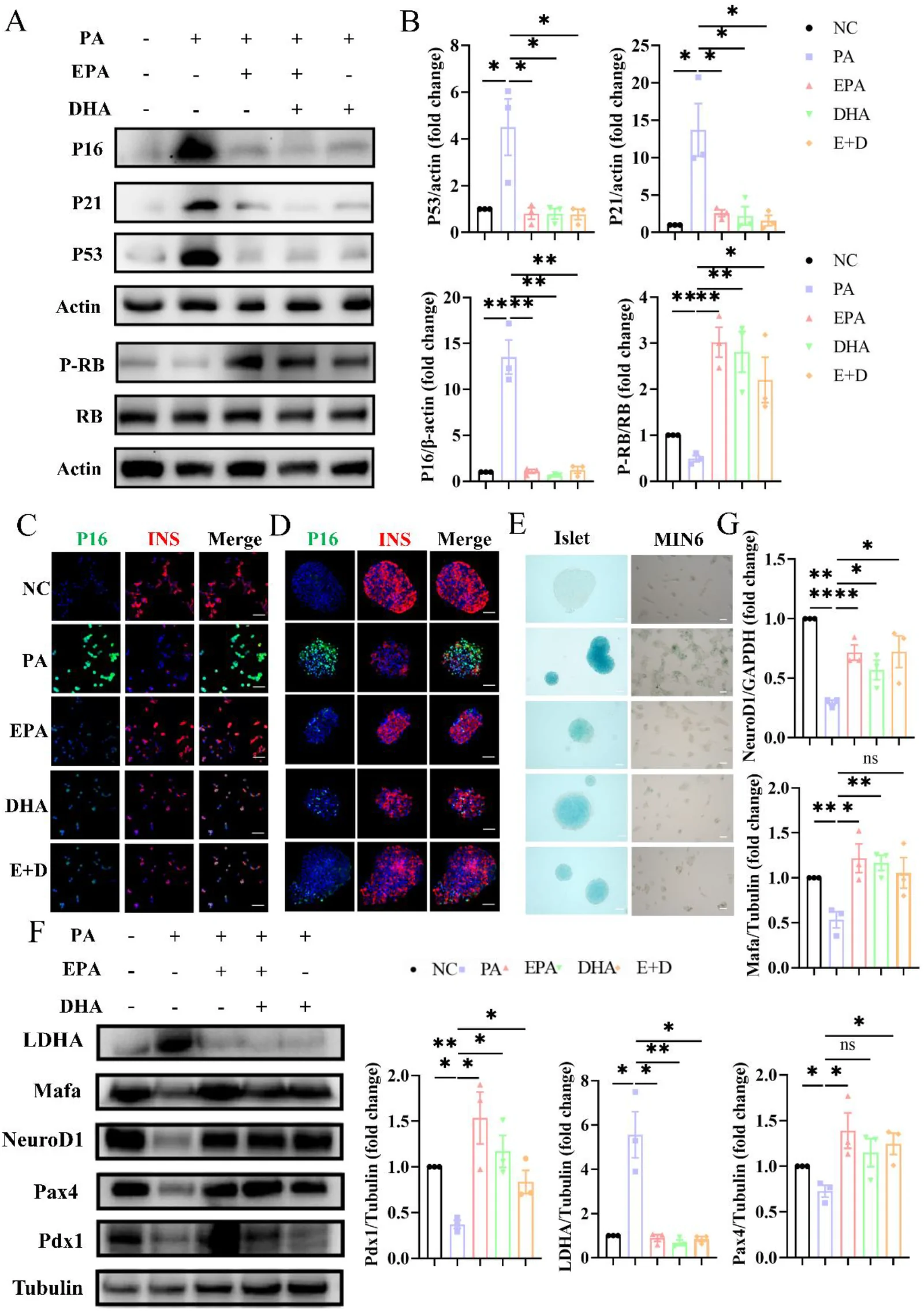
ω-3 PUFAs attenuate senescence-associated β-cell injury, inflammatory secretory signaling, and loss of β-cell identity *in vitro*. MIN6 cells and isolated mouse pancreatic islets were pretreated with EPA, DHA, or EPA+DHA (1:1) for 24 h, followed by a 24h PA (0.5 mM)/HG (25 mM) challenge in the continued presence of the corresponding treatment. **(A)** Western blot analysis of key senescence-associated proteins (P16, P21, P53, and phospho-RB) in MIN6 cells (*n* = 3). **(B)** Statistical analysis of key senescence-associated proteins (P16, P21, P53, and phospho-RB) in MIN6 cells from **(A)**. **(C, D)** Immunofluorescence co-staining of DAPI (blue), insulin (red) and P16 (green) in **(C)** MIN6 cells or **(D)** pancreatic islets. **(E)** SA-β-gal staining in MIN6 cells and pancreatic islets. **(F)** Representative western blots showing β-cell identity- and function-associated proteins, including LDHA, Mafa, NeuroD1, Pax4, Pdx1, and Tubulin. **(G)** Quantification of NeuroD1/GAPDH, Mafa/Tubulin, Pdx1/Tubulin, LDHA/tubulin, and Pax4/Tubulin. Data are expressed as the mean ± SEM from three independent biological replicates. For cell-based assays, each biological replicate represents an independently performed experiment using isolated islet preparations, as indicated. Statistical analysis was performed using one-way ANOVA followed by Sidak’s multiple-comparisons test. *P < 0.05, **P < 0.01, ****P < 0.0001. Scale bar: 50 μm.

### 3.4 ω-3 PUFAs supplementation is associated with improved glycemic indices and preservation of insulin-positive pancreatic area in HFD/STZ mice

To examine the *in vivo* phenotype associated with ω-3 PUFAs treatment, we used a HFD/STZ model combining insulin resistance with chemically induced pancreatic β cell destruction. Male C57BL/6J mice (7 weeks old) were fed an HFD (60% kcal from fat) for 4 weeks to induce insulin resistance, followed by a single intraperitoneal (i.p.) injection of STZ (100 mg/kg) to elicit β-cell injury (**Figure 5A**). Before the main experiment, we conducted a preliminary dose-finding study in the same HFD/STZ model. EPA, DHA, and E+D were administered by oral gavage at 1, 2, 3, or g·kg⁻¹·day⁻¹. During intraperitoneal glucose tolerance test (IPGTT), the 3 g·kg⁻¹·day⁻¹ dose produced the lowest glucose excursion and corresponding AUC among the doses tested for EPA, DHA, and E+D, whereas escalation to 4 g·kg⁻¹·day⁻¹ did not provide further improvement (**Supplementary Figures 1B, C**). We therefore selected 3 g·kg⁻¹·day⁻¹ for the main study. Two weeks after STZ administration, the mice with confirmed hyperglycemia (non-fasting blood glucose >16.7 mmol/L) were randomly allocated into the following groups (*n* = 6/group): a normal control (NC) group, an HFD/STZ model group administered with corn oil vehicle (H+S), and HFD/STZ groups treated with eicosapentaenoic acid (EPA, 3 g/kg/day), docosahexaenoic acid (DHA, 3 g/kg/day), or their combination (E+D, 3 g/kg/day total), all delivered daily via oral gavage for 6 months.

**Figure 5:**
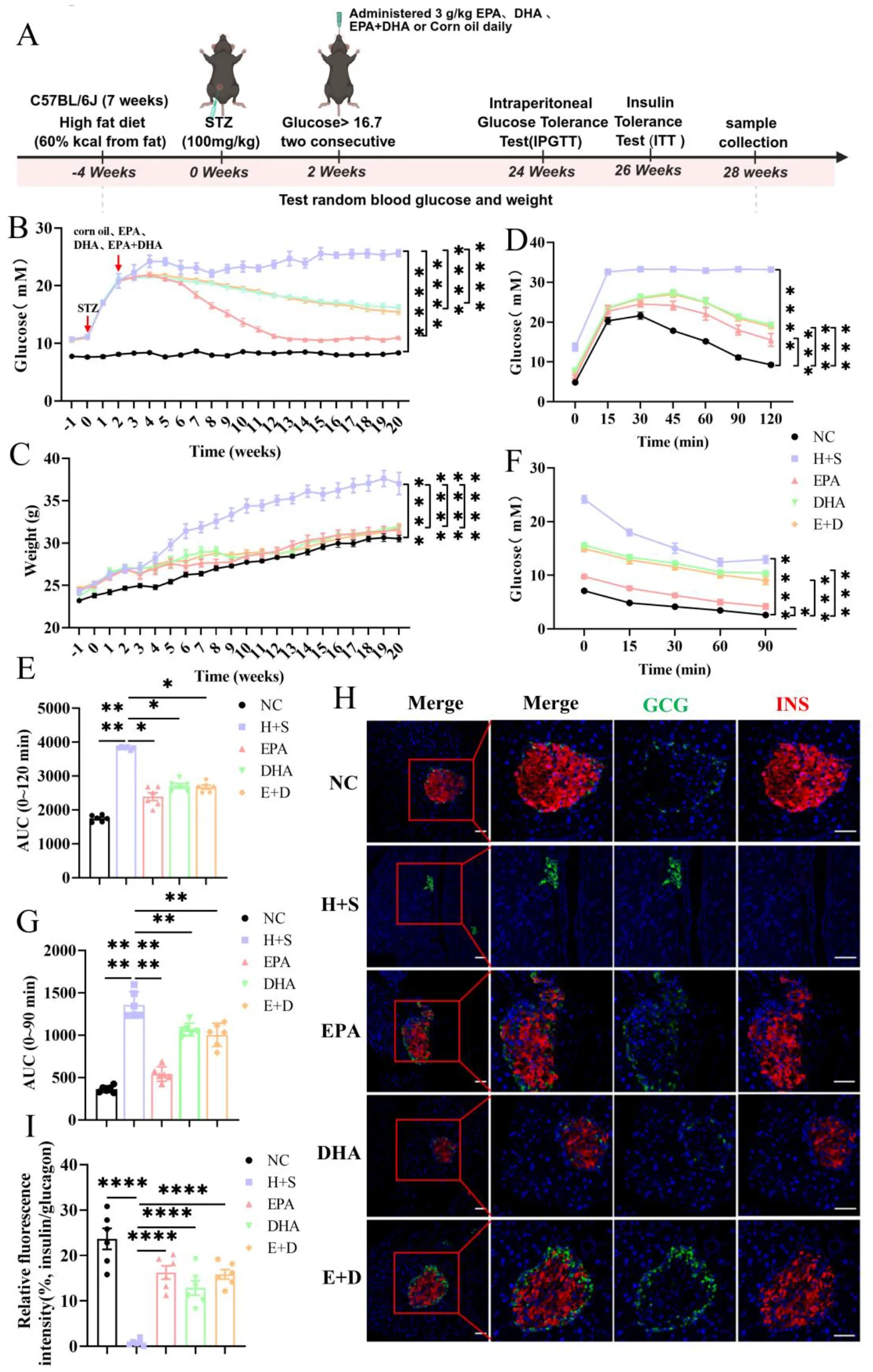
Effects of ω-3 PUFAs on glucose homeostasis in a high-fat diet/streptozotocin (HFD/STZ)-induced diabetic mouse model. **(A)** Experimental timeline and group allocation (*n* = 6 per group): normal control (NC), HFD/STZ (H+S), HFD/STZ with EPA (EPA), HFD/STZ with DHA (DHA), and HFD/STZ with combined EPA and DHA (EPA+DHA). Diabetes was induced on day 0 by an intraperitoneal (i.p.) injection of STZ (100 mg/kg). **(B)** Random blood glucose levels every week. **(C)** body weight changes during the study period. **(D)** Intraperitoneal glucose tolerance test (IPGTT) blood glucose profiles and **(E)** the corresponding area under the curve (AUC). **(F)** Insulin tolerance test (ITT) curves and **(G)** the corresponding AUC. **(H)** Representative immunofluorescence images of pancreatic islets stained for DAPI (blue), insulin (red) and glucagon (green). **(I)** Statistical analysis of insulin and glucagon co-staining shown in **(H)**. Data are presented as the mean ± SEM from three independent biological replicates. For animal-derived samples, biological replicates represent individual mouse preparations, as indicated. Statistical analysis was performed using one-way ANOVA followed by Sidak’s multiple-comparisons test. *P < 0.05, **P < 0.01, ***P < 0.001, ****P < 0.0001. Scale bar: 50 μm.

Relative to the H+S group, ω-3 PUFA-treated mice showed lower random blood glucose values during the study period (**Figure 5B**). The treated groups also had lower body weights than the H+S groups (**Figure 5C**). Furthermore, ω-3 PUFAs were associated with lower glucose excursions during an intraperitoneal glucose tolerance test (IPGTT) and a corresponding reduction in the area under the curve (AUC) (**Figures 5D, E**). Insulin sensitivity was assessed by ITT, in which the treatment groups showed lower blood glucose values and AUC during an insulin tolerance test (ITT) (**Figures 5F, G**). Immunofluorescence analysis revealed higher insulin-positive pancreatic area in the mice receiving ω-3 PUFAs supplementation (**Figures 5H, I**). Collectively, these findings show that EPA, DHA, and E+D were associated with improved glycemic indices and preservation of insulin-positive pancreatic area in this HFD/STZ model.

### 3.5 ω-3 PUFAs attenuate oxidative-stress markers and increase nuclear NRF2 staining in pancreatic islets of diabetic mice

Given the changes in oxidative-stress markers observed in the model, we next investigated whether the observed protection was linked to enhanced antioxidant defense. Indeed, the model diabetic mice exhibited reduced total antioxidant capacity (T-AOC), SOD activity, GSH content, and CAT activity, together with increased MDA levels (Yang et al., 2025) (**Figures 6A–E**). EPA, DHA, and E+D partially restored these redox measures relative to the H+S group. DCFH-DA fluorescence showed reduced islet ROS staining in the islets of ω-3 PUFAs-treated mice (**Figures 6F, I**). Immunofluorescence analysis showed an increased proportion of insulin-positive cells with nuclear NRF2 staining in the ω-3 PUFAs-treated groups (**Figures 6G, J**). These findings associate ω-3 PUFA treatment with reduced islet oxidative-stress markers and increased nuclear NRF2 localization in the HFD/STZ model.

**Figure 6:**
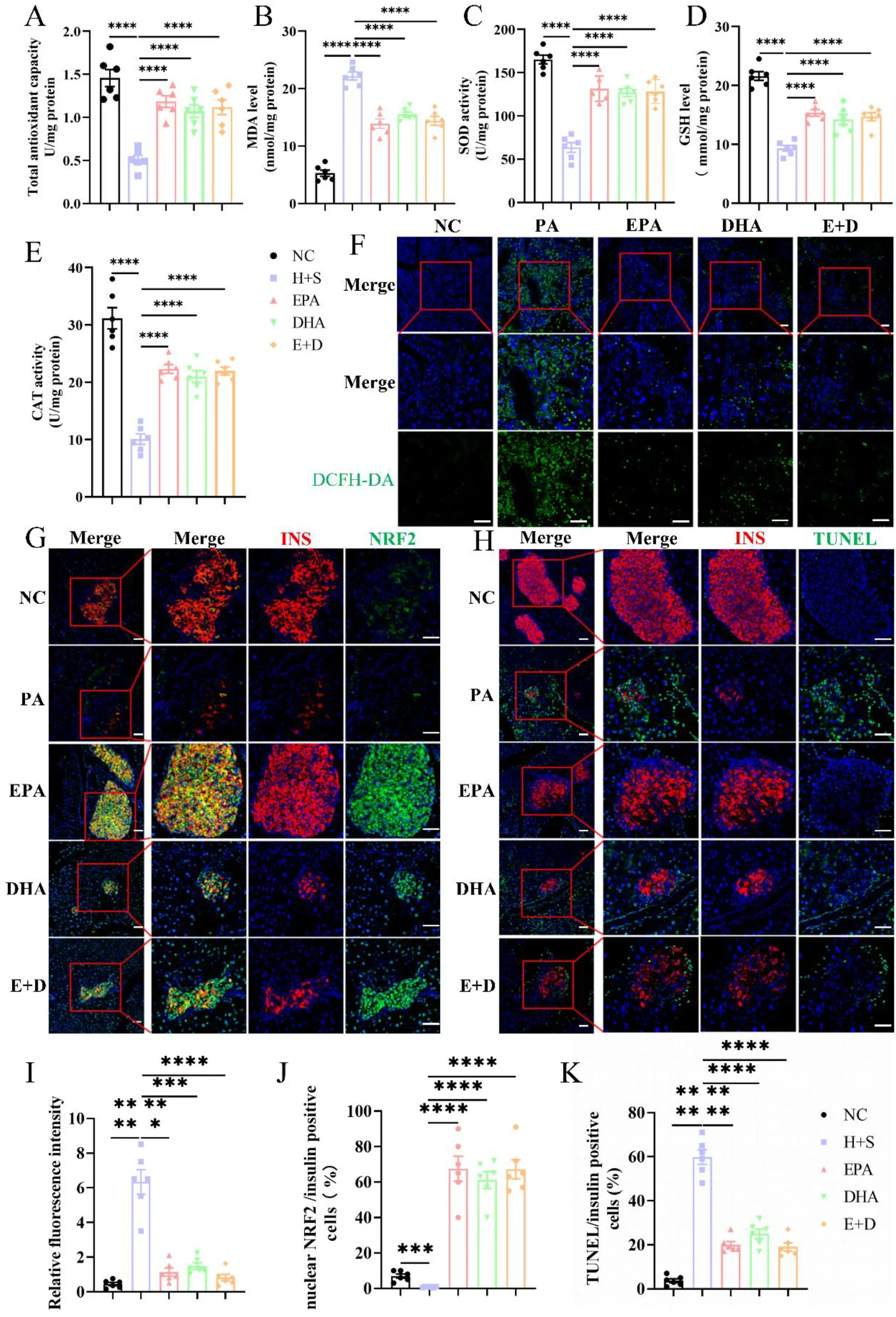
ω-3 PUFAs attenuate oxidative stress and apoptosis in pancreatic tissue of HFD/STZ-induced diabetic mice. **(A)** Total antioxidant capacity and the levels of key oxidative stress markers: **(B)** malondialdehyde (MDA), **(C)** superoxide dismutase (SOD) activity, **(D)** glutathione (GSH), and **(E)** catalase (CAT) activity (*n* = 6). **(F)** Intracellular ROS levels measured by DCFH-DA (green) fluorescence. **(G)** Representative immunofluorescence images showing DAPI (blue), NRF2 (green) localization and insulin (red) expression in pancreatic islets. **(H)** Assessment of β cell apoptosis by TUNEL (green) and insulin (red) co-staining. **(I)** Statistical analysis of ROS levels (DCFH-DA staining) from **(F)**. **(J)** Statistical analysis of nuclear NRF2 and insulin co-staining intensity from **(G)**. **(K)** Statistical analysis of TUNEL and insulin co-staining intensity from **(H)**. Data are presented as the mean ± SEM from three independent biological replicates. For animal-derived samples, biological replicates represent individual mouse preparations, as indicated. Statistical analysis was performed using one-way ANOVA followed by Sidak’s multiple-comparisons test. ***P < 0.001. ****P < 0.0001. Scale bar: 50 μm.

### 3.6 ω-3 PUFAs intervention attenuates β cell apoptosis and senescence-associated markers in diabetic mice

We next assessed apoptosis and senescence-associated markers in pancreatic tissue. TUNEL staining revealed a marked increase in apoptotic insulin-positive cells in the diabetic mice, which was significantly suppressed by ω-3 PUFAs intervention (**Figures 6H, K**). We also assessed senescence-associated markers in pancreatic islets. ω-3 PUFAs treatment effectively reduced p16 immunoreactivity (**Figure 7A**) and attenuated senescence-associated β-galactosidase activity (**Figure 7B**) in the pancreatic tissues, particularly within islet-containing regions. Together, these data show that ω-3 PUFAs treatment were associated with lower apoptotic and senescence-associated markers in pancreatic tissue.

**Figure 7:**
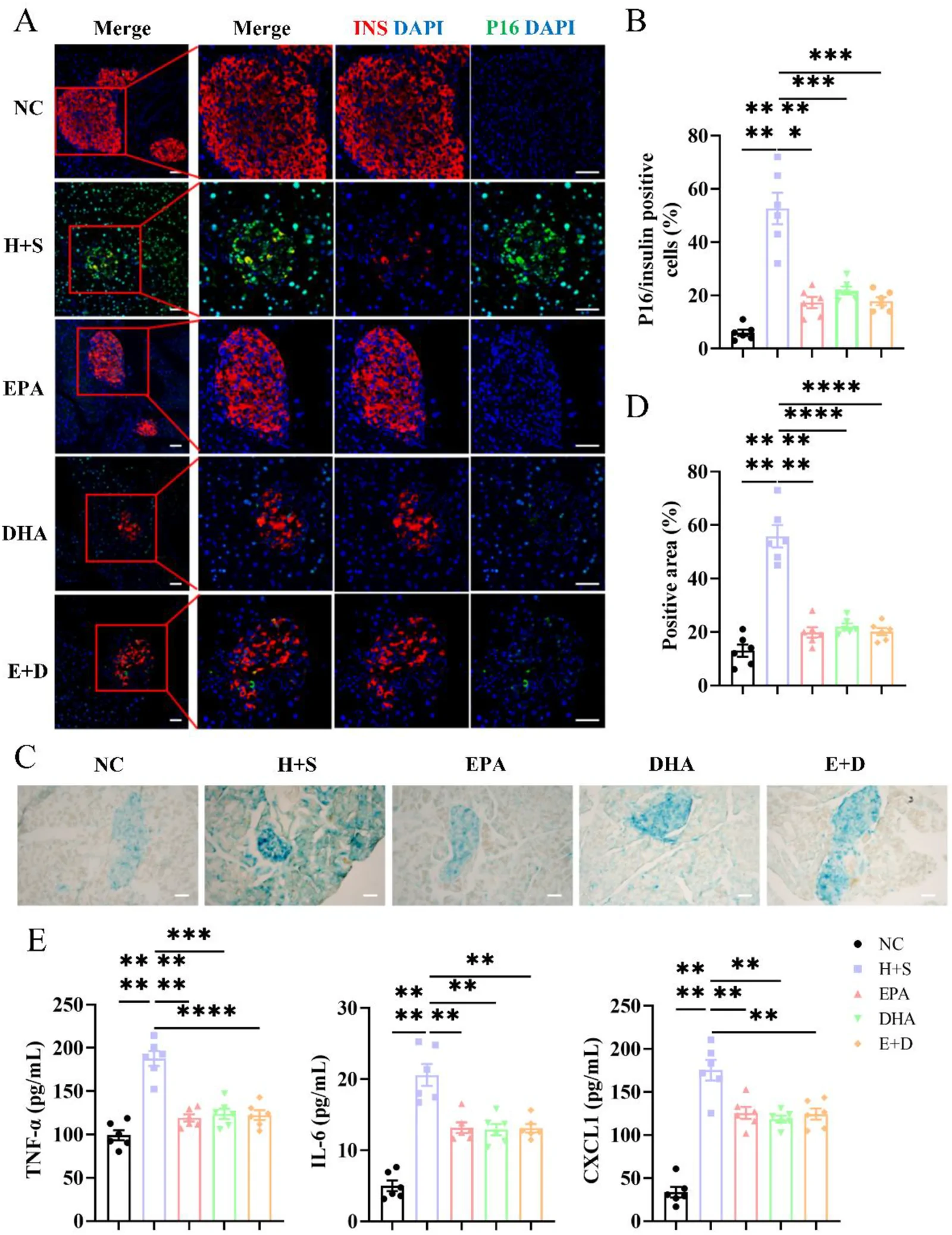
ω-3 PUFAs supplementation attenuates senescence-associated β-cell injury and inflammatory cytokine production in a diabetic model. **(A)** Representative immunofluorescence images of pancreatic islets co-stained for the senescence marker P16 (green) and insulin (red). **(B)** Quantitative analysis of P16-positive β cell area corresponding to **(A)** (*n* = 6). **(C)** Representative images of senescence-associated β-galactosidase (SA-β-gal) staining (blue) in pancreatic tissues. **(D)** Quantification of SA-β-gal positive area shown in **(C)** (*n* = 6). **(E)** Serum concentrations of TNF-α, IL-6, and CXCL1, measured by enzyme-linked immunosorbent assay. Data are presented as the mean ± SEM from three independent biological replicates. For animal-derived samples, biological replicates represent individual mouse preparations, as indicated. Statistical analysis was performed using one-way ANOVA followed by Sidak’s multiple-comparisons test. **P < 0.01, ***P < 0.001, ****P < 0.0001. Scale bar: 50 μm.

### 3.7 NRF2 knockdown attenuates the effects of ω-3 PUFAs on selected antioxidant and apoptosis-associated protein markers

To examine the contribution of NRF2 to selected molecular responses, we deployed a loss-of-function strategy in MIN6 cells. Genetic knockdown of NRF2 using specific shRNA successfully reduced its basal expression by about 70% (**Figures 1A, B**). The knockdown of NRF2 attenuated the elevation of NRF2 protein induced by ω-3 PUFAs and altered the ω-3 PUFAs-mediated suppression of its endogenous inhibitor, KEAP1, compared to those of the control shRNA-transfected cells. Importantly, in the Nrf2-knockdown cells cultured under the corresponding glucolipotoxic conditions, the ω-3 PUFAs-mediated induction of key NRF2 downstream targets—including HO-1, SLC7A11, and GPX4, was substantially attenuated (**Figures 1C, D**). These findings support NRF2 involvement in the induction of these selected antioxidant proteins. Consistent with these antioxidant findings, the ability of ω-3 PUFAs to suppress the PA/HG-associated increase in the pro-apoptotic proteins Bak and Bax was attenuated in the Nrf2-knockdown cells (**Figures 1E, F**). Thus, NRF2 knockdown weakened the effects of ω-3 PUFAs on selected antioxidant and apoptosis-associated protein endpoints in MIN6 cells.

### 3.8 NRF2 knockdown attenuates ω-3 PUFAs-associated modulation of selected stress-signaling and β-cell identity-associated proteins

We next assessed whether Nrf2 knockdown altered the effects of ω-3 PUFAs on selected stress-signaling proteins. To this end, we examined the expression of key stress-associated proteins in MIN6 cells cultured under high glucose and PA stress following genetic knockdown of NRF2. Under glucolipotoxic conditions following NRF2 knockdown. In shNC cells, ω-3 PUFAs treatment significantly reduced the protein levels of p16, p21, and p53 and increased the phospho-RB/RB ratio. NRF2 knockdown weakened these treatment-associated changes (**Figures 8A, B**). These data indicate that NRF2 contributes to the modulation of the measured p16/p21/p53/RB-associated stress-signaling proteins by ω-3 PUFAs.

**Figure 8:**
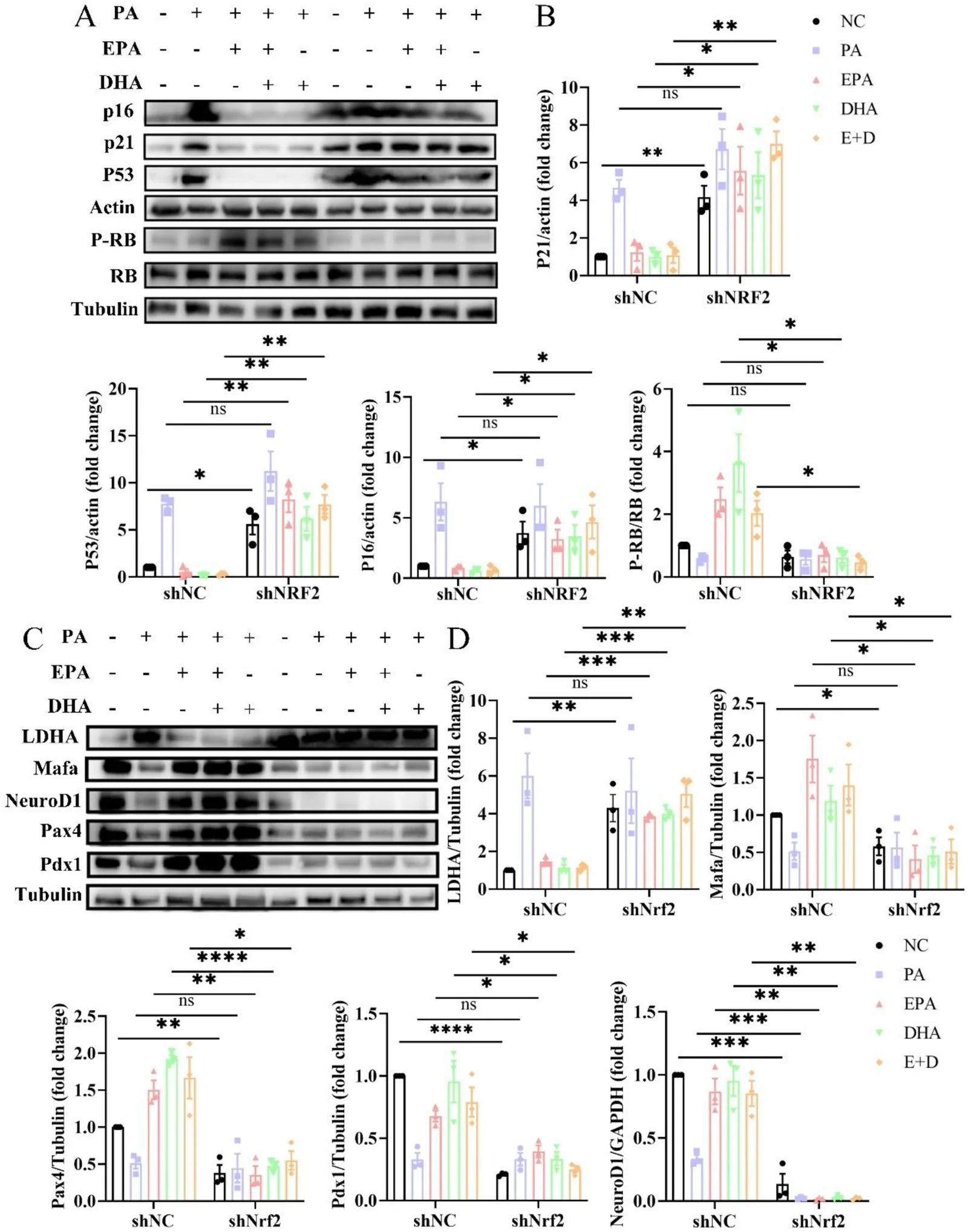
NRF2 knockdown attenuates ω-3 PUFAs associated modulation of selected stress-signaling and β-cell identity-associated proteins in MIN6 cells. MIN6 cells were transfected with control or NRF2-targeting shRNA and then treated with palmitic acid (PA, 50 μM) in the presence or absence of ω-3 PUFAs for 48 h. **(A, B)** Western blot analysis and quantification of key senescence-associated proteins (P16, P21, P53, and p-RB) (*n* = 3). **(C)** Representative western blots showing β-cell identity- and function-associated proteins, including LDHA, Mafa, NeuroD1, Pax4, Pdx1, and Tubulin. **(D)** Statistical analysis of LDHA/Tubulin, Mafa/Tubulin, NeuroD1/Tubulin, Pax4/Tubulin, and Pdx1/Tubulin. Data are expressed as the mean ± SEM from three independent biological replicates. Statistical analysis was performed using two-way ANOVA followed by Sidak’s multiple-comparisons test. *P < 0.05, **P < 0.01, ***P < 0.001, ****P < 0.0001, *ns* indicates no significant difference.

We then examined whether similar effects were observed for β-cell identity-associated proteins. In shNC cells, PA increased LDHA, a protein associated with loss of specialized β-cell metabolic features, while reducing Mafa, NeuroD1, Pax4 and Pdx1. EPA, DHA and E+D limited LDHA induction and restored the abundance of these mature β-cell identity-associated transcription factors (**Figures 8C, D**). In contrast, Nrf2 knockdown was associated with lower abundance of several β-cell identity-associated proteins, particularly NeuroD1, Pax4 and Pdx1, and attenuated the ability of EPA/DHA to reverse PA-induced LDHA elevation or restore Mafa, NeuroD1, Pax4 and Pdx1 expression.

Together, these findings show that NRF2 knockdown attenuated the effects of ω-3 PUFAs on the selected stress-signaling and β-cell identity-associated protein endpoints assessed in MIN6 cells. They do not establish that NRF2 is the sole mediator of all EPA/DHA-associated effects or that NRF2 dependency extends to unmeasured endpoints.

## 4 Discussion

β-cell survival is not synonymous with β-cell preservation. Under glucolipotoxic stress, cells that evade apoptosis may still accumulate oxidative DNA damage, sustain p53-p21 and p16-RB signaling, and lose proteins required for the mature β-cell phenotype (Gerber and Rutter, 2017; Aguayo-Mazzucato et al., 2019; Gorgoulis et al., 2019). NRF2 is already recognized as a broad regulator of β-cell fate and functional mass, with established roles in redox control, survival, adaptive proliferation, Pdx1 abundance, and insulin content under metabolic stress. Its wider roles in β-cell fate and function have also been comprehensively reviewed. Accordingly, our findings do not redefine the biological scope of NRF2. In the present acute glucolipotoxicity model, EPA and DHA reduced mitochondrial apoptotic markers, attenuated p53/p21/p16-associated stress signaling and preserved selected maturity-associated proteins. NRF2 knockdown weakened several of these molecular responses, supporting NRF2 as an important contributor to the *in vitro* response rather than its sole mediator. PA and DHA promoted NRF2 nuclear accumulation and increased the abundance of antioxidant proteins, including HO-1, NQO1, SOD1, GPX4, and SLC7A11, together with restoration of glutathione (Baumel-Alterzon et al., 2022; Patibandla et al., 2024). However, the present study did not assess ARE activity, KEAP1 modification, NRF2 ubiquitination, or NRF2 turnover; therefore, the upstream event responsible for NRF2 activation remains unresolved. The resulting reduction in lipid peroxidation and 8-hydroxy-2′-deoxyguanosine is consistent with reduced oxidative injury to membrane lipids and DNA. In other experimental systems, electrophilic oxidation products derived from EPA and DHA can modify KEAP1 and destabilize the KEAP1-CUL3 complex, thereby promoting NRF2-directed gene expression (Gao et al., 2007). This literature provides a possible biochemical explanation but does not establish that this mechanism operates in glucolipotoxic β cells.

Oxidative membrane damage may contribute to Bax/Bak associated mitochondrial outer-membrane permeabilization and apoptotic signaling, whereas persistent DNA-damage responses can sustain p53-p21 and p16-RB-associated cell-cycle checkpoint signaling (Gorgoulis et al., 2019; Di Micco et al., 2021). EPA and DHA reduced BCL-2 family imbalance, PARP cleavage, and TUNEL positivity while lowering p53, p21, p16, and senescence-associated β-galactosidase activity and restoring RB phosphorylation. These parallel changes indicate attenuation of apoptotic signaling and senescence-associated stress markers under glucolipotoxic condition, however, they do not establish a fixed causal sequence between these outcomes. Given the acute exposure paradigm, changes in p53, p21, p16, phospho-RB, and SA-β-gal activity are interpreted as attenuation of an acute stress-associated phenotype rather than as a reversal of an irreversibly established senescent state. The preservation of Pdx1, Mafa, NeuroD1, and Pax4 and the suppression of LDHA induction indicate that EPA and DHA limited glucolipotoxic disruption of a maturity-associated protein profile.Pdx1 is required for maintenance of adult β-cell identity (Gao et al., 2014), whereas depression of LDHA disrupts the specialized metabolic program that couples glucose oxidation to insulin secretion (Rutter et al., 2015; Li et al., 2026). However, these measurements do not establish preserved glucose-stimulated insulin secretion, stimulus-secretion coupling, or mature β-cell function. Dynamic insulin-secretory studies are required to define the functional consequences of the observed protein changed. PA, DHA, and their combination should not be considered interchangeable. EPA showed numerically greater protection than DHA for several endpoints, whereas EPA+DHA did not consistently exceed EPA alone. These findings suggest formulation-dependent responses, but the study was not designed to establish superiority, synergy, or antagonism among formulations. In the cell experiments, the E+D group received 25 μM EPA plus 25 μM DHA, whereas each monotherapy group received 50 μM of a single fatty acid, therefore, the influence of reduced individual EPA exposure cannot be separated from a potential EPA-DHA interaction. The molecular basis for these differences was not directly examined.

Physiologically, the primary-islet and animal experiments extend the observed protective phenotype beyond MIN6 cells. Primary mouse islets reproduced the principal antioxidant and cytoprotective responses. In HFD/STZ mice, EPA and DHA improved glycemic indices, reduced pancreatic oxidative and senescence-associated markers, lowered IL-6, TNF-α, and CXCL1, and preserved insulin-positive β-cell area and maturity-associated proteins. These *in vivo* findings demonstrate a protective phenotype in a systemic disease model, but do not establish β-cell-autonomous NRF2 causality. The HFD/STZ model incorporates peripheral insulin resistance, inflammation, altered nutrient flux, and direct STZ-induced β-cell injury, each of which may contribute to pancreatic outcomes (Winzell and Ahrén, 2004; Lenzen, 2008).

The cell-based and animal experiments address complementary experimental contexts. In cultured β cells, NRF2 knockdown attenuated selected EPA/DHA-associated molecular responses under glucolipotoxic conditions. In HFD/STZ mice, EPA/DHA administration was associated with improved metabolic indices and preservation of pancreatic markers within a systemic disease setting. Because this model includes changes in peripheral metabolism, inflammation, and direct STZ-induced pancreatic injury, the *in vivo* findings cannot distinguish β-cell-intrinsic effects from indirect systemic contributions. Accordingly, the cellular and animal data are interpreted as complementary rather than as evidence of β-cell-autonomous NRF2 causality *in vivo*.

The upstream event responsible for NRF2 activation by EPA and DHA was not investigated in the present study. In other experimental systems, electrophilic oxidation products derived from EPA and DHA have been shown to modify KEAP1 and disrupt its association with CUL3, thereby stabilizing NRF2 and promoting antioxidant-response-element-dependent gene expression (Gao et al., 2007). This mechanism provides a possible biochemical explanation for the NRF2-associated response observed here, however, its operation in glucolipotoxic β cells requires direct testing. The HFD/STZ model was used to assess EPA/DHA responses under combined diet-associated insulin resistance and β-cell stress. Although it captures relevant metabolic and pancreatic stressors, it does not reproduce the full course of human T2DM, the *in vivo* findings should therefore be interpreted within this defined preclinical context (Winzell and Ahrén, 2004; Lenzen, 2008).Treatment was initiated after sustained non-fasting hyperglycemia (>16.7 mmol/L on two consecutive assessments) had been confirmed (Sim et al., 2023), This established overt diabetes but did not quantify β-cell death at treatment initiation. Accordingly, the study cannot determine the fraction of β cells lost before treatment or infer this value from blood glucose alone.

Dose selection also limits translation. In a preliminary dose-finding experiment in the same HFD/STZ-induced C57BL/6J model, 3 g·kg⁻¹·day⁻¹ produced the lowest IPGTT excursion and AUC among the 1–4 g·kg⁻¹·day⁻¹ regimens tested, whereas 4 g g·kg⁻¹·day⁻¹ provided no additional improvement. This supported its use as an efficacy-oriented experimental dose but does not define the minimum effective dose. Body-surface-area scaling corresponds to an approximate human-equivalent exposure of 243 mg·kg⁻¹·day⁻¹, or 17 g·day⁻¹ for a 70 kg adult (Reagan-Shaw et al., 2008), substantially above the 4 g·day⁻¹ purified EPA regimen evaluated in REDUCE-IT (Bhatt et al., 2019). Together with the mixed human evidence for long-chain ω-3 PUFAs (Brown et al., 2019), these considerations indicate that the present findings demonstrate biological responses at a high preclinical exposure, not clinical efficacy, dose equivalence, or a basis for therapeutic recommendations in T2DM.

Several limitations merit explicit consideration. Only male C57BL/6J mice were studied, leaving sex-dependent responses remain unknown. Dynamic insulin secretion was not assessed, and no antioxidant comparator, such as N-acetylcysteine, was included; therefore, the relative contributions of general redox buffering versus lipid-specific EPA/DHA actions cannot be quantified. Circulating and pancreatic EPA/DHA concentrations were not measured, preventing the definition of the relationship between administered dose, tissue exposure, NRF2 engagement, and efficacy. Finally, NRF2 knockdown experiments assessed selected antioxidant, apoptotic, stress signaling, and identity-associated proteins, but did not include TUNEL, SA-β-galactosidase, insulin immunostaining, or the complete oxidative-stress panel. The knockdown data therefore support NRF2 involvement in selected *in vitro* responses, but do not establish the full spectrum of NRF2 dependence. Future rescue experiments and β-cell-specific NRF2 loss-of-function studies will be required to define pathway hierarchy and cell autonomy.

Collectively, the findings associate EPA and DHA with attenuation of glucolipotoxic oxidative injury, apoptotic signaling, and acute senescence-associated stress markers in β-cell models. The results support further investigation of NRF2-sensitive redox pathways as contributors to β-cell stress resistance, while the pathway hierarchy, functional consequences, and clinical relevance remain to be established.

## 5 Conclusion

EPA and DHA were associated with reduced glucolipotoxic oxidative injury, mitochondrial apoptotic signaling, and acute senescence-associated stress markers in β-cell models, while preserving selected maturity-associated proteins. NRF2 knockdown attenuated several of these *in vitro* responses, supporting NRF2 as an important component of, but not the sole explanation for, the EPA/DHA-associated protective phenotype. In HFD/STZ mice, ω-3 PUFAs produced beneficial systemic and pancreatic outcomes at a high experimental exposure; these findings do not establish β-cell autonomous NRF2 causality or clinical dose equivalence. Dose-ranging pharmacokinetic studies, dynamic insulin-secretory assays, antioxidant comparator arms, rescue experiments, and β-cell-specific genetic models are needed before translational conclusions can be drawn. (see **Figure 9** for the proposed model).

**Figure 9:**
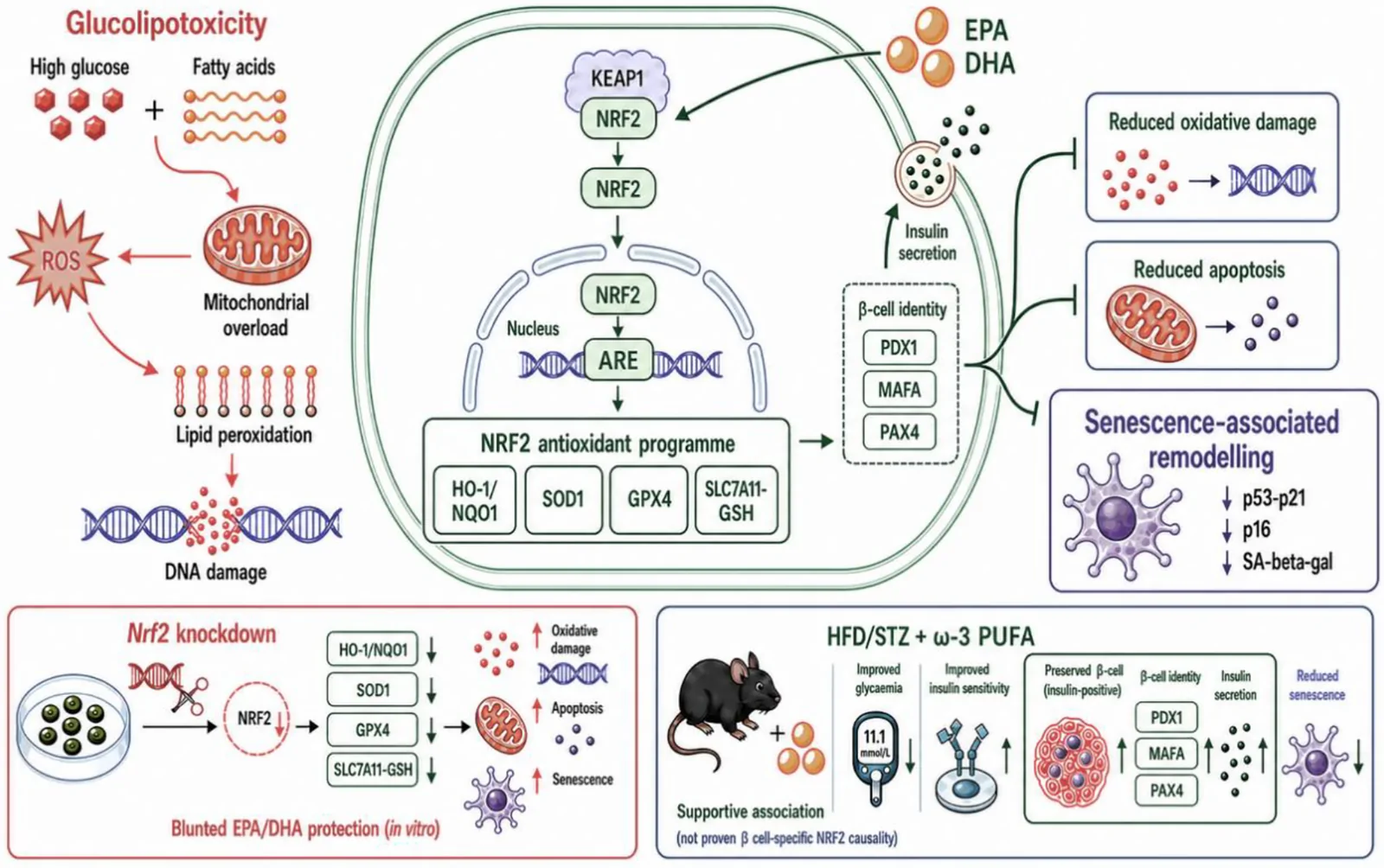
EPA and DHA are associated with NRF2-sensitive redox responses in glucolipotoxic β-cell models. EPA and DHA attenuate glucolipotoxic oxidative injury, apoptosis-associated signaling, and senescence-associated stress markers while preserving selected maturity-associated proteins in β-cell models. *NRF2* knockdown attenuates selected antioxidant, apoptotic, stress-signaling, and identity-associated molecular responses *in vitro*. In HFD/STZ mice, ω-3 PUFAs treatment is associated with improved glycemic indices and preservation of pancreatic markers, these *in vivo* findings do not establish β-cell autonomous NRF2 causality. The graphical abstract was created with BioRender.com.

## Data Availability

The raw data supporting the conclusions of this article will be made available by the authors, without undue reservation.

## Supplementary material

Supplementary Figure 1 Selection of EPA and DHA concentrations for cell experiments and doses for animal studies. **(A)** MIN6 cells were co-treated with PA (0.5 mM) and EPA, DHA, or E+D (1:1) at the indicated concentrations for 24 h, viability was assessed by CCK-8 assay (OD450). (B, C) HFD/STZ-induced diabetic C57BL/6J mice received EPA, DHA, or E+D (1, 2, 3, or 4 g·kg⁻¹·day⁻¹) by oral gavage. **(B)** IPGTT glucose curves. **(C)** IPGTT AUC. Data are mean ± SEM. Panel **(A)** was analyzed by one-way ANOVA with Sidak’s multiple-comparisons test at each concentration, panel **(B)** by two-way repeated-measures ANOVA with treatment and time as factors, followed by Sidak’s multiple-comparisons test, and panel C by one-way ANOVA with Sidak’s multiple-comparisons test. **P* < 0.05, ***P* < 0.01, ****P* < 0.001.

Supplementary Figure 2 Statistical analysis of senescence-associated signaling and SA-β-gal activity in MIN6 cells and pancreatic islets **(A)** Statistical analysis of P16 and insulin co-staining intensity in MIN6 cells from **(3D)**. **(B)** Statistical analysis of P16 and insulin co-staining intensity in pancreatic islets from **(3E)**. **(C)** Statistical analysis of SA-β-gal staining positive area in MIN6 cells **(3E)**. **(D)** Statistical analysis of SA-β-gal staining positive area in pancreatic islets **(3E)**. Data are expressed as the mean ± SEM from three independent biological replicates. statistical analysis was performed using one-way ANOVA followed by Sidak’s multiple-comparisons test. **P* < 0.05, ***P* < 0.01, ****P* < 0.001, *****P* < 0.0001.
